# Isolation of *Candida auris* from cystic fibrosis patient, Greece, April 2019

**DOI:** 10.2807/1560-7917.ES.2019.24.29.1900400

**Published:** 2019-07-18

**Authors:** Angeliki Stathi, Ioanna Loukou, Helen Kirikou, Argyri Petrocheilou, Maria Moustaki, Aristea Velegraki, Levantia Zachariadou

**Affiliations:** 1 Microbiology Department, ‘Aghia Sophia’ Children’s Hospital, Athens, Greece; 2 Cystic Fibrosis Department, ‘Aghia Sophia’ Children’s Hospital, Athens, Greece; 3 Microbiology Department, University of Athens/Hellenic Collection for Pathogenic Fungi (UOA/HCPF), Medical School, National and Kapodistrian University of Athens, Athens, Greece; 4 Mycology Department, BIOMEDICINE S.A., Athens, Greece

**Keywords:** Candida auris, Greece, first isolation, cystic fibrosis, antifungal susceptibility

## Abstract

We report the first isolation of *Candida auris* in Greece from a sputum culture of a cystic fibrosis patient in their 20s under posaconazole treatment. The pathogen was identified as *C. duobushaemulonii* by VITEK2YST, but as *C. auris* by MALDI-TOF MS*.* This case underscores the need for species-level identification of all non-*albicans Candida* (NAC) isolates from cystic fibrosis patients and patients with predisposing factors to fungal infection.

Since the first isolation of *Candida auris* from a patient with persistent fungaemia in South Korea in 1996 [1] and the first report of it being a causative agent of external otitis in Japan in 2009 [2], it has caused high-mortality invasive infections in healthcare facilities worldwide [3]. As a persistent coloniser of different human anatomical sites and an obstinate contaminant of hospital equipment and surfaces, *C. auris* spreads rapidly among critically ill patients [3-5]. We report a case where *C. auris* was isolated from a male cystic fibrosis (CF) patient in his 20s who presented with a respiratory exacerbation. To our knowledge, this is the first literature report of isolation of this emerging fungal pathogen in Greece and from a CF patient globally. This is of twofold significance as it raises concern regarding local epidemiology of *C. auris* and stresses the need for investigation of its potential interactions with other pathogenic bacteria and fungi that may affect the prognosis of CF.

## Case report

The patient, homozygous for F508del-CFTR mutation, was a student and had no history of recent travel abroad or hospitalisation. He was chronically infected with *Pseudomonas aeruginosa* and receiving long-term suppressive treatment with inhaled antibiotics, alternative cycles of tobramycin and colistin, every second month. He was also treated with lumacaftor/ivacaftor, a combination of CF transmembrane conductance regulator (CFTR) protein modulators, for more than 2 years, having no pulmonary exacerbations. Acute lung function decline in CF patients is frequently because of infections and can lead to substantial clinical deterioration.

In December 2018, because of his worsening cough and sputum production, he was diagnosed with pulmonary exacerbation and empirically prescribed ciprofloxacin for 1 month. Sputum cultures showed *Aspergillus fumigatus, Aspergillus terreus* and a non-*albicans Candida* (NAC) that was not identified to the species level. The clinical decision was to continue with the current therapeutic scheme and evaluate the patient after completion of the ciprofloxacin course. On re-examination 1 month later, cough and sputum production persisted, and his pulmonary function had deteriorated by more than 30%. Based on clinical symptoms and the results of the previous sputum culture, he was started on 300 mg oral posaconazole (three 100 mg delayed-release tablets), twice daily on the first day and 300 mg once daily thereafter. Throat swab culture taken this time grew normal flora and sputum was negative for non-tuberculous mycobacteria. After 1 month on posaconazole treatment, the patient’s spirometry was dramatically improved, and the cough and sputum production were reduced. Throat swab cultures again grew normal flora. One month later, in April 2019, the patient was still on posaconazole treatment and remained in good condition with normal spirometric values. However, this time, the sputum culture grew *P. aeruginosa, Alcaligenes denitrificans* and *C. duobushaemulonii,* as identified by VITEK2YST (bioMérieux, Marcy l'Etoile, France). Six months later, in June 2019, a new sputum culture while the patient still recieved posaconazole treatment was requested. This time, *P. aeruginosa* was isolated and posaconazole was discontinued. The patient remains in good clinical condition as at last clinical evaluation in April 2019.

## Identification of *Candida auris*

Because of the possibility of misidentifying *C. auris* [6-8], additional routine diagnostic using the MICRONAUT-Candida (MERLIN Diagnostika GmbH, Berlin, Germany) was performed. This showed an atypical profile comprising *C. famata* (probability 40.84%), *Saccharomyces cerevisiae* (probability 36.17%) and *Rhodotorulaglutinis* (probability 21.60%). Accordingly, matrix-assisted laser desorption/ionisation time-of-flight mass spectrometry (MALDI-TOF MS) (MALDI Biotyper CA System, Bruker, Billerica, Massachusetts, United States (US)) was then used and the isolate was identified as *C. auris* (score 2.00) following protein extraction [9] from 72-hour malt extract agar cultures. D1/D2 region and internal transcribed spacer (ITS) sequencing showed 99.99% homology with *C. auris*. The strain was deposited in the National and Kapodistrian University of Athens, Hellenic Collection of Pathogenic Fungi (accession number UOA/HCPF 16722), and the sequences were deposited with respective GenBank accession numbers MK975461 and MK981227.

## Antifungal susceptibility

Antifungal susceptibility by the European Committee on Antimicrobial Susceptibility Testing (EUCAST) E.Def. 7.3.1 microdilution method (January 2017) using MICRONAUT-AM Antifungal Agents MIC (MERLIN Diagnostika GmbH) showed minimum inhibitory concentration (MIC) values for amphotericin B (0.25mg/L), flucytosine (0.06 mg/L), fluconazole  (> 128 mg/L), voriconazole  (> 8 mg/L), posaconazole  (> 8 mg/L), itraconazole  (> 4 mg/L), isavuconazole (8 mg/L), micafungin (0.01 mg/L), anidulafungin (0.03 mg/L) and caspofungin (0.12 mg/L). MIC Test Strips (Liofilchem, Teramo, Italy) on RPMI agar generated results in good agreement to those observed by the microdilution method.

## Phylogenetic analysis

D1/D2-derived sequence (GenBank accession MK975461) was compared with GenBank *C. auris* D1/D2 sequences. The existing ITS GenBank sequences presented considerable heterogeneity and therefore comparison of our ITS 5/4-derived sequences are not presented. However, primary analysis (500 replicates) showed similar results to those of D1/D2 large subunit (LSU) rDNA sequences (Figure).

**Figure:**
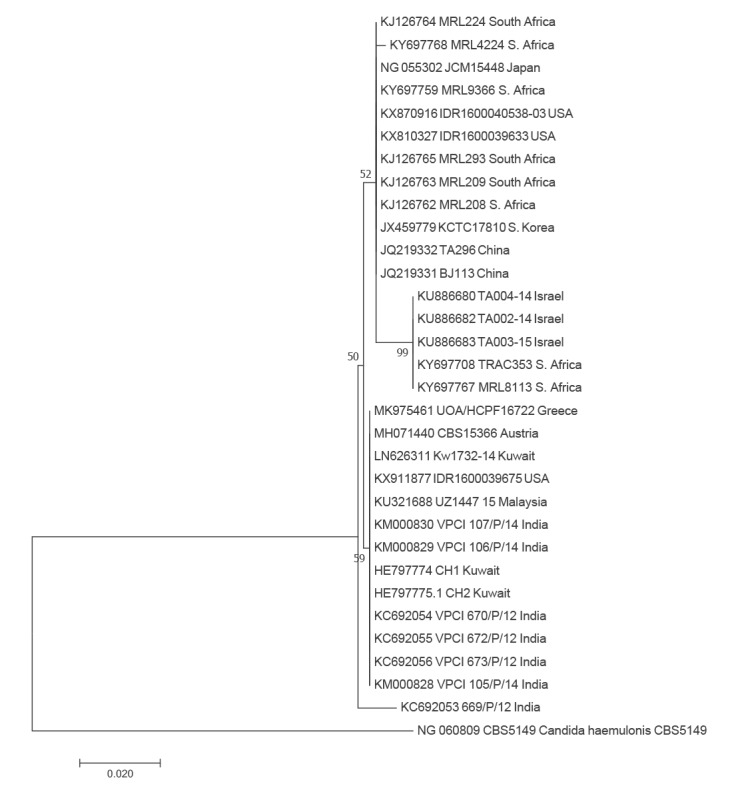
Phylogenetic tree of 500 *Candida auris* reference sequences

The phylogenetic tree was generated by neighbour-joining analysis from the partial D1/D2 large subunit (LSU) rDNA-derived sequence of *C. auris* UOA/HCPF 16722 and reference sequences. Numbers above branches are bootstrap values. The evolutionary distances were computed using the Kimura two-parameter method [16] and the robustness of the trees obtained was evaluated by 500 bootstrap replications. Alignments were constructed by ClustalW [17] and phylogenetic analysis was conducted using MEGA software version 7 [18].

The isolate in this study clusters with the south-Asian strains and is isogenic to those identified in Austria, Kuwait, Malaysia and the US. This corroborates the intercontinental spread of *C. auris* as noted by others [8].

## Epidemiological and environmental investigations

Epidemiological and environmental investigations have not yet revealed the potential source of the infection. The patient has no history of recent travel abroad or hospitalisation and to date, *C. auris* has not been isolated from any of the CF outpatients monitored at the centre visited by the patient (n = 466). All of these patients have had sputum investigated for the presence of *C. auris *since January 2019. Also 36 environmental samples taken from bed rails (n = 3), door knobs (n = 5), window sills (n = 8) and medical equipment (n = 20) of the CF outpatient centre after April 2019 were negative for *C. auris.* However, it is possible that as a student, the patient was exposed to multiple indoor and outdoor microbial environments and thus, *C. auris* may have been community acquired.

## Discussion

Healthcare services in Greece should be aware of the isolation of *C. auris* in the country. Its isolation here further demonstrates its geographical spread since 2017 [10]. Combining phylogenetic analysis with epidemiological studies would help to prevent and control the spread of *C. auris,* particularly in healthcare facilities.

In clinical laboratory practice, MALDI-TOF MS coupled with partial sequencing of selected genomic regions allows for an expeditious placement of the isolated *C. auris* in the global phylogenetic topography. However, in the clinical setting, and in the absence of MALDI-TOF MS or sequencing facilities, a promising tool for rapid routine identification of *C. auris,* is the recently validated high-throughput real-time PCR [11].

The recorded MIC values of all antifungal agents against the isolate in our study are within the ranges observed by the EUCAST microdilution method for other *C. auris* isolates [12]. The interpretive criteria based on *C. auris* MICs are currently fairly arbitrary because species-specific clinical breakpoints have not been established by EUCAST. However, the isolate’s antifungal susceptibility phenotype is typical of that reported for other *C. auris,* with amphotericin B and echinocandin low MIC values and high azole MICs [8,12].

The respiratory tract environment of individuals with CF favours polymicrobial colonisation and infection. However, the contribution of *C. auris* in poly- or mono-microbial infections and in long-term colonisation has not yet been studied in this group of patients. While the interactions of *C. albicans*, *C. glabrata*, *C. krusei* and *C. parapsilosis* with *P. aeruginosa* have been studied in the CF respiratory tract of animal models [13,14], the interactions of *C. auris,* either as a short- or long-term colonising or infective agent, with the CF respiratory tract microbial pathogens is not yet known. For this reason, it is difficult to access the potential interactions of *P. aeruginosa* and *Alcaligenes denitrificans* with the co-isolated *C. auris.* It is also difficult to evaluate whether co-isolation represented long- or short-term, possibly transient, colonisation. Further research on the interplay among the CF respiratory tract bacterial and fungal pathogens, such as *Aspergillus* spp. and *C. auris*, would be valuable.

The patient’s exacerbated condition occurred in December 2018, when two *Aspergillus* spp. and a NAC were isolated. However, as most of NAC isolates from CF specimens in our institution were not identified at the species level and because they were not stored until the end of 2018, it is impossible to confirm if the isolate in December was *C. auris*. In December 2018, the Microbiology Department at ‘Aghia Sophia’ Children’s Hospital decided that because of the global spreading of *C. auris* and sizable immigrant entry in Greece, all NAC and yeast-like fungi isolates from CF patients must be identified to species level from January 2019 forward.

Because of the patient’s inability to produce sufficient quantity of sputum, throat swabs were taken in February and March 2019 and were negative for *C. auris*.

Regarding *C. auris* isolation in April 2019, we considered two possible hypotheses on the date when the patient was infected. First, the NAC isolation in December could have been another NAC and *C. auris* colonisation started after March. Second, isolation in December could have been *C. auris* susceptible to posaconazole and under the pressure of treatment have acquired resistance or emerged heteroresistance. Consequently, the previous clinical symptoms were probably because of infection with the two *Aspergillus* species that improved on posaconazole treatment. Indeed, the high posaconazole MIC against the isolate does not exclude the possibility of a breakthrough colonisation by *C. auris*. Exacerbations in CF patients are commonly multifactorial with multiple microorganisms implicated so discerning which plays the most important role is difficult. As a result, the effect of *C. auris* on the patient’s overall clinical symptoms and course cannot be reliably assessed.

Predisposing factors to *C. auris* infection include diabetes mellitus, solid tumours, haematological malignancy, liver disease, corticosteroid therapy during hospitalisation, surgery, central venous, central nervous system (CNS) or urinary catheter, broad-spectrum antibiotics and antifungal treatment [8,15]. It is mostly isolated from bloodstream, and urinary and respiratory tract infections [7,8,15]. CF is not mentioned as a predisposing factor, but CF patients often receive antimicrobials. In addition, *C. auris* is of particular concern to treating physicians and to public health authorities as it displays resistance to many classes of antifungal agents.

Based on the isolation of *C. auris* in a CF patient, we propose that all NAC isolates also from non-sterile anatomical sites of CF patients should be identified to species level. In addition, because CF patients both undergo periods of hospitalisation and are periodically monitored at outpatient clinics, potential colonisation by *C. auris* might contribute in its dissemination within the healthcare setting and/or to other CF patients.
